# 3D Patterning of cells in Magnetic Scaffolds for Tissue Engineering

**DOI:** 10.1038/s41598-020-58738-5

**Published:** 2020-02-10

**Authors:** V. Goranov, T. Shelyakova, R. De Santis, Y. Haranava, A. Makhaniok, A. Gloria, A. Tampieri, A. Russo, E. Kon, M. Marcacci, L. Ambrosio, V. A. Dediu

**Affiliations:** 1 Institute for Nanostructured Materials, CNR-ISMN, Via Gobetti 101, 40129 Bologna, Italy; 2 BioDevice Systems, Praha 10, Vršovice, Bulharská, 996/20 Czech Republic; 3 IRCCS Istituto Ortopedico Rizzoli, Via di Barbiano 1/10, 40136 Bologna, Italy; 4 Institute of Polymers, Composites and Biomaterials, CNR-IPCB, V.le J.F. Kennedy 54 - Pad. 20 Mostra d’Oltremare, 80125 Naples, Italy; 5 Institute of Science and Technology for Ceramics, CNR-ISTEC, Via Granarolo 64, 48018 Faenza, Italy; 6 Present Address: Humanitas University Department of Biomedical Sciences, Via Manzoni 113, 20089 Rozzano, Milano, Italy; 7 Humanitas Clinical and Research Center, Via Manzoni 56, 20089 Rozzano – Milan, Italy; 8 First Moscow State Medical University (Sechenov University), Moscow, Russian Federation

**Keywords:** Cellular motility, Tissue engineering and regenerative medicine

## Abstract

A three dimensional magnetic patterning of two cell types was realised *in vitro* inside an additive manufactured magnetic scaffold, as a conceptual precursor for the vascularised tissue. The realisation of separate arrangements of vascular and osteoprogenitor cells, labelled with biocompatible magnetic nanoparticles, was established on the opposite sides of the scaffold fibres under the effect of non-homogeneous magnetic gradients and loading magnetic configuration. The magnetisation of the scaffold amplified the guiding effects by an additional trapping of cells due to short range magnetic forces. The mathematical modelling confirmed the strong enhancement of the magnetic gradients and their particular geometrical distribution near the fibres, defining the preferential cell positioning on the micro-scale. The manipulation of cells inside suitably designed magnetic scaffolds represents a unique solution for the assembling of cellular constructs organised in biologically adequate arrangements.

## Introduction

Nanotechnology and nanomaterials provide numerous innovative solutions for tissue engineering, aiming at radical reinforcement and renovation of clinical practice. The rapidly growing field of tissue engineering points at the regeneration of damaged tissues, rather than their substitution, and promotes that by implanting templates for tissue regeneration, typically represented by ceramic or polymeric scaffolds^1^. The implantation of bare cell-free scaffolds has serious limitations, especially considering the regular development of the vascular network in the regenerated tissue^2,3^. The most promising solution to this problem is the *in vitro* pre-loading of scaffolds with cells and/or growth factors before the implantation. The realisation of a correct distribution of cells inside the three-dimensional scaffolds is a key element for the maximally complete regeneration^1,4^. On the other hand, the manipulation and seeding of cells throughout the whole volume of the scaffold is an extremely challenging task and can be partially achieved via technologically sophisticated platforms^5^, barely compatible with routine clinical practice. Moreover, most tissues and organs are composed of various types of cells, preferentially arranged in complex 3D architectures, where they interact with each other and with the organism as a whole^6,7^, complicating further the requisites for a correct pre-loading. A partial solution to this challenging demand consists in the employment of the predominant cell type which defines the main tissue function^8^. This approach is generally suitable for relatively homogeneous tissues, such as cartilage, where tissue development should not be actively supported by other cell types^9^. For other tissues the achievement of efficient vascularisation, cell migration and organisation of the extracellular matrix^10^ should be necessarily supported by assembles of different cell types inside the scaffold before implantation^11^.

In this paper we propose a new, versatile and easy-to-implement method promoting the three-dimensionally controlled seeding of cells via magnetic guiding. The “naive” dragging of cells loaded with magnetic nanoparticles by external magnetic field has already been reported and succeeded in promoting solutions inaccessible to other technologies^12–17^. Here we go beyond these approaches by making use of properly designed magnetic scaffolds that are characterized by short scale (100–200 μm) strong magnetic gradients, able to orient and trap the magnetized cells on the chosen side of the scaffold fibres. This local magnetic patterning offers a handy route for the building of 3D cell architectures with a texture controlled at the microscale.

As a proof of principle of this extraordinary ability we present an accurately defined separation of two cell populations, namely mesenchymal stem cells (MSCs) and human umbilical vein endothelial cells (HUVECs), on the opposite sides of the magnetic osteogenic scaffold fibres. This combination of cells is expected to promote the reconstruction of bone microarchitecture with adequate properties, especially concerning the vascularisation of the engineered bone^18,19^. The presence of endothelial cells in tissue precursor both *in vitro* and *in vivo* prevents the central necrosis in the regenerating bone and also contributes to the maturation of the osteoblastic lineage^20,21^. Thereby, the combination of osteogenic and vasculogenic cells is known to provide a more efficient bone healing response compared to single-cell populations in different animal models^22^.

## Results

### Magnetic labelling of cells

The magnetisation of cells with commercial Chemicell fluorescent MNPs (100 nm) was based on the previously established protocol^23^, adjusted to the specific needs of our experiments. HUVECs were magnetized with nano-screen MAG/R-PAA nanoparticles with red fluorescence label, while MSCs were magnetized with nano-screen MAG/G-PAA nanoparticles with green fluorescence label.

In the first step we defined the optimal incubation time for the uptake of nanoparticle by cells. The experiment was performed at fixed 100 pg/cell concentration of nanoparticles in the cell growth medium and the incubation time was set in the range from 2 h to 84 h. The uptake of magnetic nanoparticles by cells was investigated by flow cytometry^24^, in which the side scattered signal (SSC) depends on the density and granularity of the investigated object.

The Beckman Coulter Epics Altra flow cytometer was used for a rough estimation of the MNPs concentration in cells by analysing the forward scatter and side scatter signals and cell fluorescence.

The side scatter values from the raw data of the untreated control cells was set to 100% and the side scatter increase of the nanoparticle-treated cells was calculated accordingly. Each sample was measured for the fixed time of 60 seconds.

The gating logic consisted of counting cells within a large SSC (log) vs. FCS (linear) cytogram and it was used to eliminate the small debris particles from the histograms. The scatter and fluorescent histogram parameters were derived from this gated scatter region. Most of the samples contained minimal debris with low influence on the mean of the histogram distribution.

Electronic compensation was used to eliminate the bleed through fluorescence. The data analysis was performed with Kaluza software version 2.0 (Beckman Coulter). The flow cytometry analyses were conducted in three independent experiments, each with triplicate samples.

**Figure 1:**
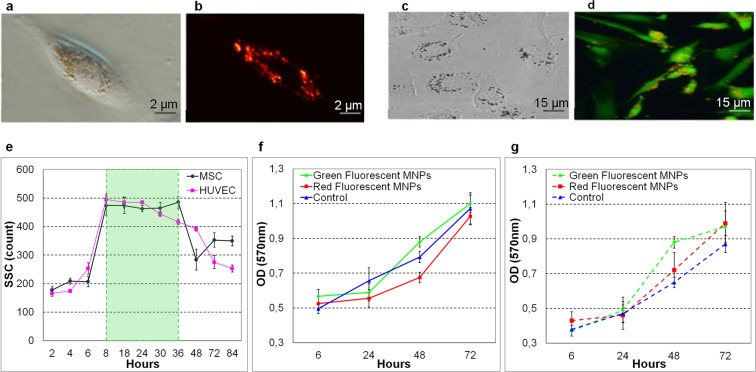
Microscopic imaging of magnetized cells (**a–d): (a)** Light microscopy of HUVEC; (**b**) Fluorescent microscopy of HUVEC; (**c**) Light microscopy of MSCs; (**d**) Fluorescent microscopy of MSCs; (**e**) Average SSC signal intensity of MSCs (circles) and HUVECs (squares) for 100 pg/cell concentration of MNPs, where the selected area indicates the stability interval; (**f**) The viability of magnetically labelled MSC cells according to MTT test; (**g**) The viability of magnetically labelled HUVEC cells according to MTT test.

The optical microscope examination (Fig. 1a–d) included both direct and fluorescence visualisation, revealing clusters of magnetic nanoparticles internalized into inner space of cells, and more specifically accumulated in perinuclear space. Typically, one cell contained clusters of different sizes (Fig. 1a,c). During the first hours after the MNPs introduction (mainly for the first 8 h), residual clusters of nanoparticles were also observed on the cell surface (Fig. 1d). These residuals were further absorbed by cells at incubation times corresponding to stability interval and typically disappeared after 18 h. For a better definition of the distribution of MNPs in MSCs, these cells were additionally stained with calcein (green fluorescence).

In our case the SSC intensity (Fig. 1e) reflected the concentration of nanoparticles in the cells^25^ and the data were averaged over six experimental sets (p < 0.05). For incubation times shorter than 4 h the level of SSC did not change significantly, undergoing a sharp enhancement at 8 h. This was followed by the 8–36 h interval corresponding to high and relatively constant concentration of MNPs (green rectangle in Fig. 1e), defining the incubation time range for the most stable magnetisation. The SSC level decreased then rapidly after 36 h, probably due to cell proliferation and the dilution or redistribution of the magnetic component.

Based on the results of the flow cytometry the 18 h incubation time was selected as optimal, considering the magnetisation strength and stability. Keeping this time fixed, different concentrations of MNPs were used for cell magnetisation (see below).

Finally, we tested the viability of both cell types in a standard approach (n = 6, p < 0.05), namely by detecting the optical density (OD) of the formazan. Noteworthily, the viability of both MSC and HUVEC was not affected by MNP concentrations as high as 100 pg/cell for periods as long as 72 hours (Fig. 1f,g). Moreover, previous studies showed that for similar magnetisation approaches, there was no adverse effect on the cell plasticity and cell phenotype for osteogenic (17 days^26^) and chondrogenic (21 days^27^) differentiation.

### Magnetic scaffolds

The cells were magnetically labelled by using commercial MNPs, while the scaffolds were projected and fabricated on the basis of advanced magnetic materials, mixing bioresorbable Fe-doped hydroxyapatite (FeHA) to the poly(ε-caprolactone) (PCL)^28^. Specifically, the 3D scaffolds were manufactured by injecting/extruding and depositing the fibres along specific directions according to the defined lay-down pattern^29,30^. The nanocomposite pellets were heated to the temperature of 110–130 °C using a cartridge unit placed on the mobile arm of a 3D Bio-Printer (Envisiontec GmbH, Gladbeck, Germany).

### Magnetic motility of cells

The magnetic force acting on the cells depends on their magnetisation and on local magnetic field gradient^31^. In order to provide similar conditions for cell manipulation and attachment to the scaffold, it is important to achieve similar magnetically driven forces for the large majority of cells. A standard magnetic characterisation of the magnetised cells is not an easy task, due to the difficulties to maintain cell-friendly conditions inside magnetometers or susceptometers. Therefore, we developed a test based on the cell motility in applied magnetic field.

**Figure 2:**
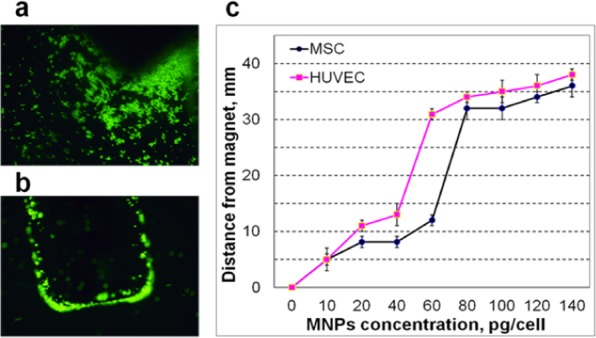
Accumulation of magnetically labelled MSCs by magnet (fluorescent microscopy, magnification 10X): (**a**) starting time, (**b**) final cell arrangement; (**c**) Motility threshold as function of MNP concentration (n = 6, p < 0.01).

The cell motility in response to magnetic guiding was initially tested qualitatively by using a cylindrical NdFeB permanent magnet with 1.2 T magnetic remanence. The magnet was placed under the bottom of the cultural dish and nearly all the cells undergone the magnetic trapping, leaving a negligible number of cells out of magnetic influence (Fig. 2a,b).

To assess quantitatively the motility response of magnetized cells to magnetic fields we employed an original approach (Fig. 2c), based on the detection of the maximal distance between magnet and cells at which the cell motion was initiated. We define by “motility threshold” the highest distance between the magnet edge and the position of cell at which the cell motion is observed. The experiment was performed on a 96-well plate filled with magnetically labelled cells. Figure 2c shows the motility threshold as function of MNP concentration used for incubation. The motility strongly increased for concentrations higher than 40 pg/cell for HUVEC and 60 pg/cell for MSC. On the basis of these data and discussed above viability, we select the 100 pg/cell concentration as most suitable, combining high motility and viability for both types of cells. Noteworthily the same concentration was widely used by other groups^32^ facilitating thus the comparison of our results with available literature data. It is important to mention that at this concentration nearly 100% of labelled cells moved at 30 mm distance from magnet edge.

**Figure 3:**
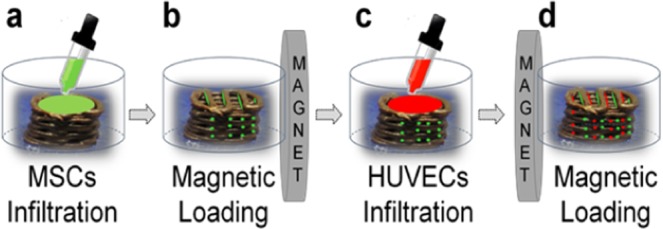
Experimental setup for 3D assembling of magnetically labelled cells inside the magnetic scaffold.

The cell seeding procedure, which represents the main experimental result of this research, is shown schematically in Fig. 3. The dry scaffold was placed in a Petri dish and infiltrated in the first step with magnetically labelled MSCs suspended in solution (concentration 10^5^ ml^−1^) (Fig. 3a). After that, the permanent magnet disc (diameter 80 mm, height 10 mm) was placed at 17 mm distance from the scaffold, while its vertical position was set in the centre-to-centre configuration (Fig. 3b), activating the magnetic loading. The loading was applied for 3 hours, during which strong magnetic gradients moved the cells inside the scaffold, so that the cells were directed towards the fibre side opposite to the magnet. In the second step, the same procedure was performed with magnetically labelled HUVECs in a fully reversed magnetic field configuration (Fig. 3c,d).

**Figure 4:**
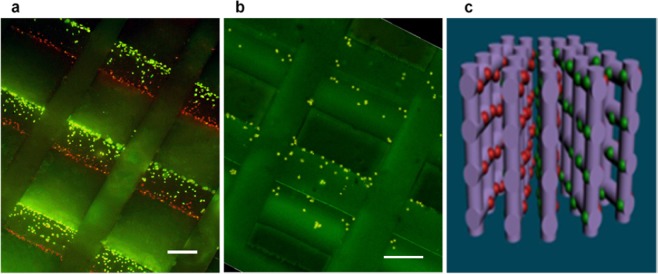
Micro-spatial patterning of magnetically labelled cells in magnetized scaffolds (bar 200 µm): (**a**) magnetic PCL/FeHA scaffold; (**b**) non-magnetic PCL/HA scaffold; (**c**) schematic illustration of magnetically assembled 3D cellular architectures.

The proof of concept result of our research is shown in Fig. 4. The employed magnetic loading allowed to establish two separated and well organized cell colonies inside the magnetic scaffold (Fig. 4a). Each magnetic fibre was similarly covered by the two cell populations, organized into green (MSCs) and red (HUVECs) assemblies on the opposite sides of fibres. Cell layers were arranged in regular structures determined by the inner microarchitecture of scaffold. Each layer dominantly contained cells of one type, with a negligible admixture with the second type. A small number of cells with visually insufficient magnetic labelling (poor visible fluorescence) remained unattached to the scaffold surface.

Evidently the cell population was established only on fibres perpendicular to the magnetic field. The cells did not attach to those fibres where the magnetic forces caused cell motion along the fibres, preventing the possibility for the cell to stop and adhere to the surface. The population of those fibres would have required the second loading step with the rotation of scaffold by 90°. We intentionally avoided this step to emphasize the versatility and selectivity of the proposed cell loading.

MTT assay was routinely employed to evaluate the cell viability. A 20 µl of 3,4,5- dimethylthiazolyl-2,5-diphenyl tetrazolium bromide (MTT, Sigma) dye solution (5 mg/mL in medium) was added to the medium containing cells. After 4 h of incubation at 37 °C, the medium was removed, and Formazan crystals were dissolved in 200 µl of dimethylsulphoxide (DMSO) and quantified by measuring the absorbance of the solution at 570 nm by a microplate reader (FACSstat 3200). All analyses were conducted in three independent experiments, each with 6 samples. Importantly, MTT test confirmed that up to 48 hours after the magnetic loading the viability and proliferative activity of HUVECs/MSCs cell system on investigated magnetic scaffolds was similar to the that of the control batch (scaffold free, cells in monolayer culture).

The cell seeding in magnetic scaffolds was benchmarked against non-magnetic scaffold case, processed in the same conditions: 5 non-magnetized and 5 magnetized scaffolds were used in each experimental series. Non-magnetic scaffolds presented visibly lower accumulation and attachment of magnetized cells on the fibre surface (Fig. 4b). For simplicity we have used only MSCs (green) cells for the non-magnetic scaffolds. The cells attached on the fibres of non-magnetic scaffold had also a different, less homogeneous, distribution compared to the magnetic scaffold.

Even though the long-term development of cells inside magnetic scaffolds was not directly related to the scope of this research, the viability of loaded cells inside the scaffolds was monitored by MTT tests up to 72 hours. It was confirmed that the viability/proliferative activity of HUVECs/MSCs in investigated magnetic scaffolds was closely comparable to the control monolayer mixed-cell culture. Moreover, tissue-typic tubular-like structures have developed on scaffold fibres surfaces covered with HUVECs, indicating that the scaffold environment does not reduce the vasculogenic ability of cells.

Summarizing, the intended 3D architecture combining 2 different cell types inside specially designed magnetic scaffolds was successfully realized (Fig. 4a,c) as a potential precursor for vascularized tissue engineering.

### Computer simulations

To understand and quantify the effect of the scaffold magnetisation on the organisation of magnetized cells, the finite element simulation was carried out. Computer modelling with COMSOL 3.5 was employed to calculate the magnetic field and its gradient distributions in the Multiphysics – Magnetostatics – 2D axial symmetry application mode.

**Figure 5:**
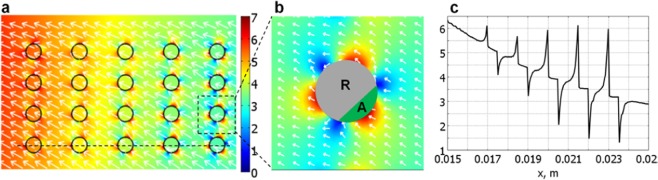
Magnetic gradient in T/m units: (**a**) distribution in the scaffold region: colour scale indicates the magnitude while arrows show the direction of gradient and hence the direction of magnetic forces; (**b**) zooming around one fibre section, where green area delineates the attraction region (A), and grey area indicates the repulsion region (R); (**c**) gradient distribution along the dashed line in (**a**).

The scaffold magnetisation was taken from the experimental curve for PCL/FeHA(80/20) published previously^29^ and it was assumed that the fibres were uniformly magnetized with the saturation magnetisation of about 1 emu/g. The chosen geometry reproduced most important parameters of the experimental scaffolds and consisted of 0.5 mm diameter coaxial cylindrical fibres arranged so that their axial plane corresponded to a square lattice of 5 × 4 elements with a pitch of 1 mm horizontally and 0.5 mm vertically (Fig. 5a). A NdFeB permanent magnet disc was placed similarly to the experimental settings (see Fig. 4) but, in order to use the axial symmetry mode significantly simplifying the simulations, the magnetic disc was shifted vertically with respect to the experimental set-up, keeping the bottoms of magnet and scaffold on the same level.

The strength and direction of driving magnetic forces are defined by magnetic gradients and by cell magnetisation^31^. We focused our simulations on the calculation of magnetic gradients. The Fig. 5 shows the quantitative distribution of the magnetic gradients inside the whole scaffold (a), locally around one selected fibre (b), and along the line crossing a complete raw of fibres (c).

The modelling revealed that the magnetic scaffold acts as a local non-linear modulator of the background magnetic field and gradient generated by the permanent magnet. While the general direction of the cell motion is defined by the position of the external magnet (Fig. 5a), a particular magnetic configuration was unravelled closely around the fibres. The combination of local areas around the magnetic fibres alternating high and low gradient values (6 T/m in red spots and 1 T/m in blue spots) yields to the formation of attractive and repulsive fibre regions, as depicted by arrows in Fig. 5a,b. This generates a specific magnetic configuration leading to a strongly preferential seeding of cells, in good agreement with cellular patterns observed experimentally (Fig. 4a).

The magnetic scaffolds significantly promote thus the formation of ordered, heterogeneous cellular structures and the detected non-linear effects considerably modify the magnetic guiding and trapping with respect to non-magnetic scaffolds. Noteworthily, as evidenced in Fig. 5c, the modulation of the background gradients caused by sole magnet are even higher at longer distances.

## Discussion

It is well known that an appropriate micro-spatial distribution of different cell populations in 3D constructions is one of the most important challenges of tissue engineering. Such distribution is essential not only for the development of fully functional tissue but also to ensure the faster vascularisation.

Standard surgical intervention involving introduction of scaffolds into the organism may cause a distortion of the microenvironmental cues^33^. Also, inflammatory processes can promote the development of fibrous connective tissues (cicatrisation) with a low level of functionality, complicated further by hypoxia and preventing the formation of a complete vascular network^34^. Therefore, the early engraftment of the implant with sufficient vascularisation is the prerequisite for comprehensive integration with the host tissue. This is especially important for the substitution of large bone fragments, where it is necessary to minimize the risk of hypoxic areas formation to avoid further biomechanical insufficiency^33,34^.

The field has indeed been widely investigated by the scientific community and numerous solutions have been advanced based on various nanotechnology platforms and microfluidic devices^35,36^. Nevertheless many issues have yet to be solved, especially considering the possibility to transfer these technologies to the surgery practice. Most of the micro-scale cell patterning methods require a close influence of a manipulator on separate cells or on the small homogeneous groups of cells^37^. In particular, organ printing uses layer-by-layer deposition of cells or matrix^38^. Lithographic methods and photo-patterning require essential modification of scaffold substrate^39–42^. The abovementioned methods use the consequent capture and fixation of cellular elements in a single layer with a further “set” of these layers to form three-dimensional tissue-like structures. In all the cases, this requires considerable time and precise integration between the cell layers. Table 1 reassumes most diffused methods and describes their advantages and disadvantages^36^, while the last column describes the proposed in this paper technology.

**Table 1:** Benchmarking magnetic technology versus basic 3D cell printing approaches.

	Laser-assisted^45,46^	Inkjet^47–49^	Microextrusion^50–52^	Magnetic assembling
Advantages	(1) Single cell manipulation (2) Nozzle free (3) Usage of high viscosity bio-ink (4) High resolution (5) High accuracy (6) High gelation speed	(1) High cell viability (2) Noncontact nozzle (3) Printed cell patterns using different cell types (4) Heterogeneous multicellular constructs (5) High throughput (6) High gelation speed	(1) High mechanical properties (2) Short fabrication time (3) Printing of various types and viscosities of bio-ink (4) Wide range of biocompatible materials	(1) High cell viability (2) Remote control of cell distribution in deep scaffold space (3) Simultaneous assembling of large number of cells (5) Simple and rapid methodology (6) Heterogeneous multicellular constructs
Disadvantages	(1) Low mechanical properties (2) Long fabrication time (3) Damage cells due to heat generated from laser energy (4) Aggregate in the final tissue construct	(1) Low mechanical and structural integrity (2) Long fabrication time (3) Low upper limit for viscosity of bio-ink (4)Low reproducibility (5) Cell aggregation (6) Clogging of the nozzle orifice	(1) Low cell viability due to nozzle wall shear stress and mechanical stress (2) Low accuracy (3) Cell death due to changes in dispensing pressure and bio-ink concentration	(1) Magnetic labeling of cells is mandatory (2) Spatial control over cells distribution is established only for large ensembles, and not for a single cell (3) Spatial distribution strongly depends on the scaffold geometry and magnetization

The magnetic 3D patterning of cells has thus a great potential for the development of functionally adequate and efficient implants. The performed separation of the two cell types (see Fig. 4) was intentional with the separation distance mimicking biological requirements. Indeed, for both *in vitro* and *in vivo* cases the distance between cells and capillaries able to provide nutrients and oxygen and take care of waste elimination does not exceed 200 µm^43^. The presented here distribution of cells and their multilayer interposition is a prerequisite for most efficient development of the vascular tree. The achieved cellular organisation in the scaffold fulfils also the conditions for the development of tissue precursors with appropriate heterogeneous structure. To preserve this cellular organisation, arranged with the purpose to support regular tissue structures, we foresee the necessity to implant the scaffold within a few hours after loading.

The advanced here technology is simple and employs a remote control for cell distribution in deep scaffold space. Differently from all other methods, it allows to manipulate simultaneously a large number of cells. Moreover, a relatively small amount of nanoparticles (100 pg/cell) is sufficient to provide an effective multilayered scaffold loading without detectable losses of the cell viability.

The simulations unveiled quite unusual and not yet fully exploited properties of the magnetic scaffolds. The attractive regions play the double role of positioning the cells and of pinning them for a time sufficient to bind to the fibre, resulting in an ordered structure from a higher number of cells with respect to the non-magnetic scaffolds. It is important to note that even relatively low magnetisation of the scaffold (less than 1 emu/g) produces local forces strong enough to attract the cells towards the closest fibre, significantly contributing to the cell adhesion. Moreover, in our simulation we used a homogenous magnetisation of fibres, while the magnetic non-uniformity at the microscopic level would further increase the local gradients and lead to a higher pinning of cells near the fibres^29,30^.

Summarizing, we established and validated a handy and versatile magnetic bio-technology able to generate on-demand complex 3D cellular architectures in implantable scaffolds. It represents a prototype on an innovative 3D cellular printer based on remote magnetic control, while its validation *in vivo* should necessarily be tailored to the specific biological situation under investigation^44^. We believe that this technology provides unavailable so far routes and means for the achievement of cell populations and distribution inside the tissue regeneration implants. Moreover, we do not see real obstacles for the utilisation of the developed technology for *in vivo* environment, even though possible upgrades and adaptations may be required.
